# Eyelid ptosis and muscle weakness in a child with Kawasaki disease: a case report

**DOI:** 10.1186/s12887-021-02979-4

**Published:** 2021-11-27

**Authors:** Yao Lin, Lijun Wang, Aijie Li, Hongwei Zhang, Lin Shi

**Affiliations:** Department of Pediatric Cardiology, Children’s Hospital, Capital Institute of Pediatrics, No. 2 Yabao Road, Chaoyang District, Beijing, 100020 China

**Keywords:** Kawasaki disease, Ptosis, Muscle weakness, Myositis, Case report

## Abstract

**Background:**

Kawasaki disease (KD) is an acute febrile vasculitis that often occurs in children under 5 years. Ptosis and muscle weakness associated with KD are rarely documented.

**Case presentation:**

We present a case of KD with eyelid ptosis and muscle weakness in a 3-year-old boy. At admission, grade IV and grade III muscle strength were recorded for upper and lower limbs, respectively. Diminished patellar tendon reflex was noted. Laboratory evaluation showed hypokalemia with the serum potassium concentration of 2.62 mmol/L. Intravenous immunoglobulin (IVIG) and aspirin were initiated immediately accompanied with methylprednisolone for adjunctive therapy. Potassium supplement was administered at the same time, which resulted in the correction of hypokalemia on the 2nd day of admission but no improvement in ptosis and muscle weakness. Neostigmine testing, lumber puncture, electromyography, and cerebral and full spine MRI were performed, which, however, did not find evidence for neural and muscle diseases. On the 5th day, the fever was resolved. On the 6th day, eyelid ptosis disappeared. And on the 14th day, the muscle strength and muscle tension returned to normal, patellar tendon reflex could be drawn out normally, and the boy regained full ambulatory ability.

**Conclusions:**

KD might affect the neural and muscular systems, and KD complicated with eyelid ptosis and muscle weakness is responsive to the standard anti-inflammatory treatment plus adjunctive corticosteroid therapy.

## Article summary

A 3-year-old boy with typical Kawasaki disease also presenting with eyelid ptosis and muscle weakness was treated with a standard anti-inflammatory regimen plus adjunctive therapy in acute stage and recovered completely.

## Background

Kawasaki disease (KD) is an acute febrile vasculitis that often occurs in children under 5 years. Coronary artery lesions are the most common complications of KD. Neural system complications are uncommon, which mainly include febrile convulsion, aseptic meningitis and auditory nerve palsy [1]. Eyelid ptosis and muscle weakness, especially the simultaneous presence of these two conditions in acute phase of the disease, are rarely documented. In this article, we present a case of eyelid ptosis and muscle weakness secondary to KD.

## Case presentation

**Fig. 1:**
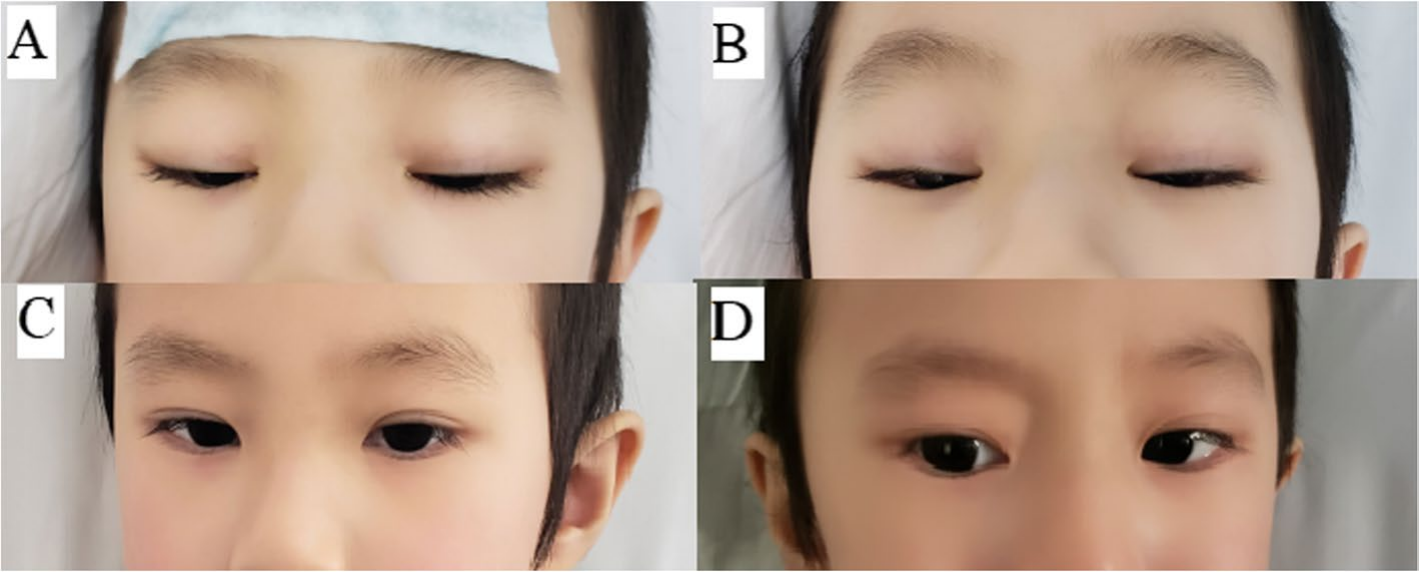
The time course of eyelid ptosis in a 3-year-old boy with KD. **A** the 1st day of admission; **B** the 3rd day of admission; **C** the 6th day of admission; and D: the 14th day of admission

A 3-year-old boy with fever for 5 days was admitted to our hospital. KD was diagnosed by typical symptoms including rashes, strawberry tongue, cervical lymphadenitis, conjunctivitis, and extremity edema. Besides these symptoms, the boy was noted to have eyelid ptosis (Fig. 1), muscle weakness with diminished patellar tendon reflex, nasal congestion and mastoid tenderness. The muscle strength of upper limbs was scaled as grade IV, and lower limbs grade III. Blood testing was performed, which revealed increased white blood cell counts, elevated C-reactive protein and transaminases, and decreased K^+^ (Table 1). Echocardiography showed normal bilateral coronary artery but minor regurgitation in the mitral and tricuspid valves. Cranial CT suggested otitis media and mastoiditis. As COVID-19-associated multisystem inflammatory syndrome overlaps with KD, the patient underwent testing for COVID-19. Both RT-PCR and antibody measurement were negative. Intravenous immunoglobulin (IVIG) with the dosage of 2 g/kg and aspirin with the dosage of 30 mg/kg/d were given immediately. Furthermore, according to the Kobayashi risk stratification [2], methylprednisolone infusion was initiated (2.8 mg/kg/d, administered every 8 h) for adjunctive anti-inflammatory therapy. Other therapies were administered at the same time, including latamoxef recommended by an otorhinolaryngologist for otitis media and mastoiditis, human albumin infusion, potassium supplement, and glutathione (reduced) and glycyrrhizin for hepatoprotection.

**Table 1:** Blood testing results at admission

Parameter	Result
White blood cell count	22.48 × 10^9^/L
Neutrophil ratio	97%
C-reactive protein	173.89 mg/L
Procalcitonin	17.67 ng/ml
Erythrocyte sedimentation rate	82 mm/h
Alanine transaminase	101.7 U/L
Aspertate aminotransferase	42.1 U/L
Total bilirubin	33.2 μmol/L
Direct bilirubin	23.6 μmol/L
K^+^	2.62 mmol/L
Na^+^	128 mmol/L
D-Dimer	2 mg/LFEU
Serum albumin	24.4 g/L
Creatine kinase	62 U/L
Blood gas analysis	metabolic acidosis

On the 2nd day of admission, the hypokalemia and hypoalbuminemia were corrected with the serum concentration of 3.89 mmol/L and 31.7 g/L respectively. However, fever, eyelid ptosis and muscle weakness were not improved. Family history of disease with similar symptoms was denied and toxin exposure was excluded. Neostigmine testing, lumber puncture, and cerebral and full spine MRI were performed, which, however, did not show evidence for neural and muscular diseases such as myasthenia gravis, Guillain-Barre syndrome and meningitis. On the 5th day of admission, the fever was resolved, and the lower limb muscle strength was recovered gradually to grade IV with a weak patellar tendon reflex elicited. Nasal congestion and mastoid tenderness were improved gradually. On the 6th day of admission, eyelid ptosis disappeared; ophthalmoscopy, electromyography and metabolism disease screening showed normal results; white blood cell count and C-reactive protein became normal; and aspirin was reduced to 3 mg/kg daily. On the 8th day of admission, 5 days after the initial KD rashes disappeared, the boy started to develop new itching rashes over his trunk, and the blood routine showed elevated eosinophil counts. Drug allergy was suspected, and aspirin and latamoxef were replaced by clopidogrel and ertapenem respectively. Cetirizine was administered for anti-allergy therapy. The rashes faded 3 days later. Methylprednisolone dosage was reduced to 1 mg/kg/d and tapered in 4 weeks (completed in outpatient clinic). On the 14th day of admission, the muscle strength and muscle tension returned to normal completely, patellar tendon reflex could be drawn out normally, and the boy regained full ambulatory ability. Laboratory evaluation showed normal hepatic and renal function, stable electrolytes, and normal D-Dimer. Echocardiography showed normal size and function of the heart without any regurgitation and normal inner diameter of bilateral coronary artery. Hearing screening was normal. Whole-exome sequencing did to identify genetic abnormalities. The patient was discharged after 20 days of hospitalization. At the 1-month follow-up after discharge, the boy was healthy without muscle weakness and eyelid ptosis recurrence.

## Discussion and conclusion

Eyelid ptosis or muscle weakness associated with KD is uncommon. We present here a case of concurrent eyelid ptosis and muscle weakness in the acute stage of KD, an even rarer entity. A singular neurological symptom associated with KD has been described in several case reports [3–10]. Simultaneous presence of ptosis and limb muscle weakness in a patient with KD is novel. Koutras reported that an 18-month-old girl with KD had proximal muscle weakness, dysphonia and dysphagia on the 8th day of illness [3]. Although laboratory examination showed normal creatine kinase levels, electromyography revealed myositis. Myositis associated with KD eventually diagnosed by electromyography was also described in 2 other cases [4, 5]. Additionally, 4 cases of myositis concurrent with KD were confirmed by muscle biopsy [4–7]. We tried to identify the potential cause for ptosis and muscle weakness in our case. However, no elevated serum creatine kinase was detected and no muscle damages were observed; results of neurological exams were normal and genetic cause was also excluded. It is unlikely that hypokalemia caused ptosis and muscle weakness in our patient as correction of hypokalemia did not result in improvement of ptosis and muscle weakness.

Despite the confirmatory diagnosis of myositis in KD reported previously, the mechanism of myositis is unresolved. We speculated that vasculitis around the affected muscle and nerve might be the culprit, but why only palpebralis and limb muscles were affected remains unknown. Further study is warranted, which, however, is challenging due to the rarity of the condition.

Different treatment methods have been applied for ptosis secondary to KD. Zhao et al. treated a 5-year-old girl with typical KD, and the patient developed ptosis 3 days after IVIG administration. After a series of examinations, oculomotor nerve palsy was diagnosed. The child was given aspirin and prednisone, and recovered 4 weeks later with symptoms of ptosis beginning to relieve 3 weeks after the initiation of aspirin and prednisone [11]. Thapa et al. reported that two boys with KD who developed right-side oculomotor nerve palsy that was manifested by ipsilateral ptosis and medial rectus palsy recovered 5 days after IVIG treatment [12]. Another study showed that a patient with KD complicated with orbital cellulitis had eyelid movement restriction that regressed 3 days after aspirin administration [13]. Hameed et al. reported that a 3-year-old boy developed bilateral ptosis on day 21 (5 days after IVIG administration), which resolved on day 25 without any other special therapies [14]. In the present case, the patient received IVIG, aspirin and methylprednisolone for the management of KD complicated with eyelid ptosis and muscle weakness, and the outcome is satisfactory. These data indicate that in addition to the standard anti-inflammatory treatment, adjunctive therapy might be required for the effective management of eyelid ptosis and muscle weakness associated with KD, which is helpful for clinicians working in the field.

In conclusion, KD might affect the neural and muscular systems, and KD complicated with eyelid ptosis and muscle weakness is responsive to the standard anti-inflammatory treatment plus adjunctive corticosteroid therapy.

## Data Availability

The data of this case are available from the corresponding author on reasonable request.

## Abbreviations

KD: Kawasaki disease
IVIG: Intravenous immunoglobulin
